# Comparisons of heart rate variability responses to head-up tilt with and without abdominal and lower-extremity compression in healthy young individuals: a randomized crossover study

**DOI:** 10.3389/fphys.2023.1269079

**Published:** 2024-01-08

**Authors:** Kazuaki Oyake, Miyuki Katai, Anzu Yoneyama, Hazuki Ikegawa, Shigeru Kani, Kimito Momose

**Affiliations:** Department of Physical Therapy, School of Health Sciences, Shinshu University, Matsumoto, Japan

**Keywords:** autonomic nervous system, postural orthostatic tachycardia syndrome, rehabilitation, tilt-table test, RR interval (RRI)

## Abstract

**Introduction:** Abdominal and lower-extremity compression techniques can help reduce orthostatic heart rate increases. However, the effects of body compression on the cardiac autonomic systems, which control heart rate, remain unclear. The primary objective of this study was to compare heart rate variability, a reflection of cardiac autonomic regulation, during a head-up tilt test with and without abdominal and lower-extremity compression in healthy young individuals. The secondary objective was to conduct a subgroup analysis, considering participant sex, and compare heart rate and heart rate variability responses to head-up tilt with and without compression therapy.

**Methods:** In a randomized crossover design, 39 healthy volunteers (20 females, aged 20.9 ± 1.2 years) underwent two head-up tilt tests with and without abdominal and lower-extremity compression. Heart rate and heart rate variability parameters were measured during the head-up tilt tests, including the Stress Index, root mean square of successive differences between adjacent R-R intervals, low- and high-frequency components, and low-to-high frequency ratio.

**Results:** Abdominal and lower-extremity compression reduced the orthostatic increase in heart rate (*p* < 0.001). The tilt-induced changes in heart rate variability parameters, except for the low-frequency component, were smaller in the compression condition than in the no-compression condition (*p* < 0.001). These results were consistent regardless of sex. Additionally, multiple regression analysis with potentially confounding variables revealed that the compression-induced reduction in Stress Index during the head-up tilt position was a significant independent variable for the compression-induced reduction in heart rate in the head-up tilt position (coefficient = 0.411, *p* = 0.025).

**Conclusion:** Comparative analyses revealed that abdominal and lower-extremity compression has a notable impact on the compensatory sympathetic activation and vagal withdrawal typically observed during orthostasis, resulting in a reduction of the increase in heart rate. Furthermore, this decrease in heart rate was primarily attributed to the attenuation of cardiac sympathetic activity associated with compression. Our findings could contribute to the appropriate application of compression therapy for preventing orthostatic tachycardia. This study is registered with UMIN000045179.

## 1 Introduction

Postural orthostatic tachycardia syndrome (POTS), a chronic form of orthostatic intolerance, primarily affects women of child-bearing age. POTS involves symptoms such as dizziness, palpitations upon standing, and a heart rate increase of 30 beats per min (bpm) or more, without orthostatic hypotension (Freeman et al., 2011; Sheldon et al., 2015; Arnold et al., 2018). In addition, POTS is often associated with symptoms of depression, anxiety, sleep disturbances, cognitive dysfunction, exercise intolerance, functional disability, and impaired health-related quality of life (Bagai et al., 2011; Shibata et al., 2012; Anderson et al., 2014; Pederson and Brook, 2017; Hutt et al., 2020; Rich et al., 2022; Vas et al., 2022). Abdominal and lower-extremity compression garments have been used as a potential treatment for POTS (Heyer, 2014; Bryarly et al., 2019; Bourne et al., 2021).

Assuming an upright position increases gravitational forces, leading to blood pooling, estimated at 500–800 mL, in the venous system, pelvic and splanchnic circulation, and lower extremities. This pooling reduces venous return, stroke volume, and mean blood pressure. The subsequent compensatory response involves sympathetic activation and vagal withdrawal, resulting in increased heart rate, cardiac contractility, and peripheral vascular resistance (Mosqueda-Garcia et al., 2000). External compression applied to the abdomen and/or lower extremities can help redirect pooled blood back to the heart and potentially reduce the orthostatic increase in heart rate (Watanuki and Murata, 1994; Dorey et al., 2018; Lee et al., 2018; Kelly et al., 2019). The effects of compression on orthostatic changes in hemodynamic variables, such as heart rate, blood pressure, stroke volume, cardiac output, and total peripheral resistance have been investigated in healthy adults (Watanuki and Murata, 1994; Platts et al., 2009; Stenger et al., 2010; Stenger et al., 2013; Dorey et al., 2018; Lee et al., 2018; Kelly et al., 2019), individuals with orthostatic hypotension (Denq et al., 1997; Smit et al., 2004; Podoleanu et al., 2006), and those with POTS (Heyer, 2014; Bourne et al., 2021). However, to the best of our knowledge, no study has compared orthostatic changes in heart rate variability parameters with and without compression as a main goal, even in healthy individuals.

Analysis of heart rate variability is widely used as a standard method for assessing cardiac autonomic nervous functions controlling heart rate (Camm et al., 1996; Laborde et al., 2017; Shaffer and Ginsberg, 2017). Therefore, the primary objective of this study was to compare heart rate variability responses to head-up tilt (HUT) with and without abdominal and lower-extremity compression in healthy young individuals. Previous studies have shown that increases in heart rate and decreases in heart rate variability parameters were associated with vagal withdrawal during the initial transition from supine to standing positions in healthy controls (Stewart, 2000; Freitas et al., 2015; Orjatsalo et al., 2020; Ali et al., 2021). Based on existing knowledge, we hypothesized that abdominal and lower-extremity compression would reduce the orthostatic decrease in vagally mediated heart rate variability parameters and the subsequent increase in heart rate in comparison to no compression.

Previous studies investigating the effectiveness of compression in reducing orthostatic tachycardia among individuals with POTS have primarily included female participants, accounting for more than 90% of the sample (Heyer, 2014; Bourne et al., 2021). This sex imbalance has limited the generalizability of the findings to males. Studies indicating that compression garments mitigated orthostatic heart rate increases in healthy adults had an almost equal distribution of male and female participants (Dorey et al., 2018; Lee et al., 2018; Kelly et al., 2019). However, an analysis stratified by sex was not performed in these studies. Thus, the secondary objective of our study was to conduct a subgroup analysis to compare heart rate and heart rate variability responses to HUT with and without compression garments based on participant sex.

## 2 Materials and methods

### 2.1 Study design

This study used a randomized crossover design. Participants underwent two consecutive HUT tests under no-compression and compression conditions. A 10-min supine rest period was designated between each test as a washout period. Participants were randomly assigned to either the no-compression or compression condition in the first trial using computer-generated random numbers. The study protocol was approved by the appropriate ethics committee of Shinshu University (approval number: 5254). All participants provided written informed consent before enrolment in the study. The study was performed per the 1964 Declaration of Helsinki, as revised in 2013.

### 2.2 Participants

Recruitment was conducted by placing posters on the university campus. The inclusion criteria comprised age of 20–40 years, absence of underlying diseases, no history of syncope, and no smoking habit. The exclusion criteria were as follows: limited range of motion and/or pain that affects the HUT test, medical compression treatment contraindicated (Rabe et al., 2020), and taking any medication that interferes with cardiovascular control.

### 2.3 Abdominal and lower-extremity compression

In the compression condition, participants were outfitted with an inflatable abdominal band along with medical compression stockings. The inflatable abdominal band consisted of an outer band made of hard polyester cloth with Velcro straps and an inner inflatable cuff of an aneroid sphygmomanometer (Tanaka et al., 1997). The band was attached to the participants around the lower abdomen between the pubis and umbilicus (Smit et al., 2004; Figueroa et al., 2015) and inflated to 35–45 mmHg to compress the abdominal wall. The medical compression stockings used in this study (Jobst Bellavar class 3; BSN-JOBST GmbH, Emmerich am Rhein, Germany) delivered approximately 35–45 mmHg of pressure at the ankle. Appropriately sized stockings were selected according to the circumference of the ankle and calf, as recommended by the manufacturer.

### 2.4 HUT test

Two trained assessors performed the HUT tests in a quiet room at a comfortable temperature. Participants were asked to refrain from eating and consuming caffeinated products for at least 2 h and to avoid vigorous physical activity for at least 12 h before each test (Cheshire and Goldstein, 2019; Finucane et al., 2019). The tests were performed between 5:00 and 7:00 p.m.

In the HUT test, participants remained in a resting supine position on a motorized tilt table (TB-653; Takada Bed Manufacturing Co., Ltd., Osaka, Japan) for 5 min before postural change. After the 5-min supine rest, the tilt table was elevated to an angle of 70° for approximately 30 s and was maintained for 10 min (Cheshire and Goldstein, 2019; Bourne et al., 2021). If a participant experienced a severe symptom, such as presyncope, the test was promptly halted, and the participant was repositioned to a supine position. Throughout the HUT tests, participants were instructed to regulate their breathing at a rate of 0.25 Hz (approximately 15 breaths per minute) using a computer metronome. This measure was implemented to minimize any influence of respiratory fluctuations on heart rate variability assessments (Schipke et al., 1999; Laborde et al., 2017). The audio cue was available to help participants pace their breathing.

### 2.5 Measurements and data analysis

The R-R intervals, the time interval between two consecutive R waves in the electrocardiogram, were continuously recorded during the HUT test using a portable 3-lead electrocardiograph monitor (Active Tracer AC-301A; GMS Inc., Tokyo, Japan) with a sampling rate of 1 kHz. Blood pressure was measured every minute on the left arm using an automated sphygmomanometer (HEM-907; Omron Co., Ltd., Kyoto, Japan). At the end of each test, participants were instructed to report the severity of orthostatic symptoms, such as dizziness and palpitations, that occurred during 10 min of HUT on a visual analog scale (VAS). The possible score ranged from 0 (no symptoms) to 100 (syncope or presyncope), measured in millimeters on a 100-mm horizontal line using a pen.

Heart rate and heart rate variability parameters during the supine period were determined from the R-R intervals recorded during the 5-min supine rest period, while those during the HUT period were derived from the last 5 min of the HUT period. A threshold-based artifact correction algorithm with a medium filter was applied to discriminate and remove ectopic beats and artifacts (Baker et al., 2018a; Baker et al., 2018b). The medium filter identified all R-R intervals that were longer/shorter than 0.25 s compared to the local average. The correction was made by replacing the identified artifacts with interpolated values using a cubic spline interpolation. These calculations were performed using Kubios HRV standard software version 3.5 (Kubios Oy, Kuopio, Finland). The root mean square of successive differences between adjacent R-R intervals (RMSSD) was analyzed as the time-domain measure of heart rate variability to estimate vagal activity (Camm et al., 1996; Laborde et al., 2017; Shaffer and Ginsberg, 2017). Furthermore, the Stress Index, which is the square root of Baevsky’s stress index (Baevsky and Chernikova, 2017), serves as an indicator of sympathetic activity (Ali et al., 2021; Kubios HRV Software, 2021). Baevsky’s stress index was calculated using the following formula:

$$\text{Baevsky}’s\text{ stress index}={\text{AM}}_{O}*100*{\left(2M_{O}*M_{X}{\text{DM}}_{n}\right)}^{-1}$$

Where mode (M_o_) is the most frequent R-R interval expressed in seconds. The amplitude of the mode (AM_o_) was determined by employing a 50-ms bin width, quantified as the percentage of R-R intervals within the bin containing the mode in relation to the total number of R-R intervals recorded. Variation range (M_x_DM_n_) represents the disparity between the longest (M_x_) and shortest (M_n_) values of R-R intervals, measured in seconds. Enhanced sympathetic activation of the heart leads to a more stable heart rhythm, resulting in an increased occurrence of R-R intervals with similar durations. Thus, when cardiac sympathetic activity increases, the histogram of R-R intervals becomes narrower and increases in height, leading to a higher Stress Index. Additionally, as power spectral analysis of the R-R intervals has also been widely used to quantify cardiac autonomic regulation (Camm et al., 1996; Laborde et al., 2017; Shaffer and Ginsberg, 2017), the frequency-domain parameters of heart rate variability were calculated using the fast Fourier transform model. Fast Fourier transform analysis used a Welch periodogram method with a window width of 256 s and 50% window overlap. Low-frequency (0.04–0.15 Hz) and high-frequency (0.15–0.40 Hz) spectral components were collected in absolute values of power (ms^2^). The low-frequency heart rate variability (LF) component reflects a complex and not easily discernible mix of sympathetic, parasympathetic, and other unidentified factors (Billman, 2013). The high-frequency heart rate variability (HF) component reflects vagal tone and is more affected by respiratory rate than RMSSD (Laborde et al., 2017; Shaffer and Ginsberg, 2017). Furthermore, the LF/HF ratio was calculated from the absolute values of LF and HF. The LF/HF ratio is often regarded as an indicator of the sympathetic-parasympathetic balance, despite receiving some criticism for this interpretation (Billman, 2013).

The averaged values of blood pressure variables during the supine period were calculated based on data collected during the 5-min supine rest period, while those during the HUT period were derived from the final 5 min of the HUT period. The changes in hemodynamic and heart rate variability parameters from supine to HUT were calculated by subtracting the values measured during the supine position from those obtained during the HUT position.

### 2.6 Statistical analysis

The G Power computer program version 3.1.9.2 (Heinrich Heine University, Dusseldorf, Germany) (Erdfelder et al., 1996) was used for sample size calculation to detect differences in hemodynamic and heart rate variability responses to HUT between the no-compression and compression conditions. Based on a previous study that compared hemodynamic responses to HUT with and without lower-extremity compression in healthy adults (Lee et al., 2018), we used an estimated effect size of 0.80 for the paired t-test. Considering a statistical power of 0.80, an alpha level of 0.05, and an effect size of 0.80, the estimated sample size required was 15. Thus, to facilitate subgroup analysis comparing heart rate and heart rate variability responses to HUT between the no-compression and compression conditions based on participant sex, a minimum of 15 males and 15 females was deemed necessary.

Hemodynamic and heart rate variability parameters were subjected to a two-way repeated-measures analysis of variance (ANOVA) with two positions (supine and HUT) and two conditions (no compression and compression) as within-subject factors. A paired t-test with Bonferroni correction was performed to compare hemodynamic and heart rate variability parameters between the conditions in each position. In the subgroup analyses by sex, the changes in heart rate and heart rate variability parameters from supine to HUT were compared with and without compression using a paired t-test. Additionally, the VAS scores between the conditions were compared using the Wilcoxon signed-rank test.

To explore the heart rate variability parameters associated with the compression-induced reduction in standing heart rate, we calculated the difference in heart rates during the HUT with and without compression as the dependent variable. Regarding the heart rate variability parameters with a significant interaction between position and condition, we also calculated the differences in the measurements during the HUT position with and without compression as the independent variables. The correlations between the dependent and independent variables were determined using Pearson’s product-moment correlation coefficient. In addition, to identify potential confounding variables, we examined the associations of the compression-induced change in heart rate with age, height, weight, body mass index, and the changes in systolic and diastolic blood pressure during the HUT position with compression using Pearson’s product-moment correlation coefficient and unpaired t-test based on variable types. We performed multiple regression analysis with forced entry to confirm whether the associations between the changes in heart rate and heart rate variability parameters observed in the bivariate analysis remained significant, even when adjusting for potential confounding variables. Statistical analyses were performed using GraphPad Prism version 9.00 for Windows (GraphPad Software, San Diego, California, United States). Statistical significance was set at a *p*-value less than 0.05.

## 3 Results

### 3.1 Participants

**FIGURE 1:**
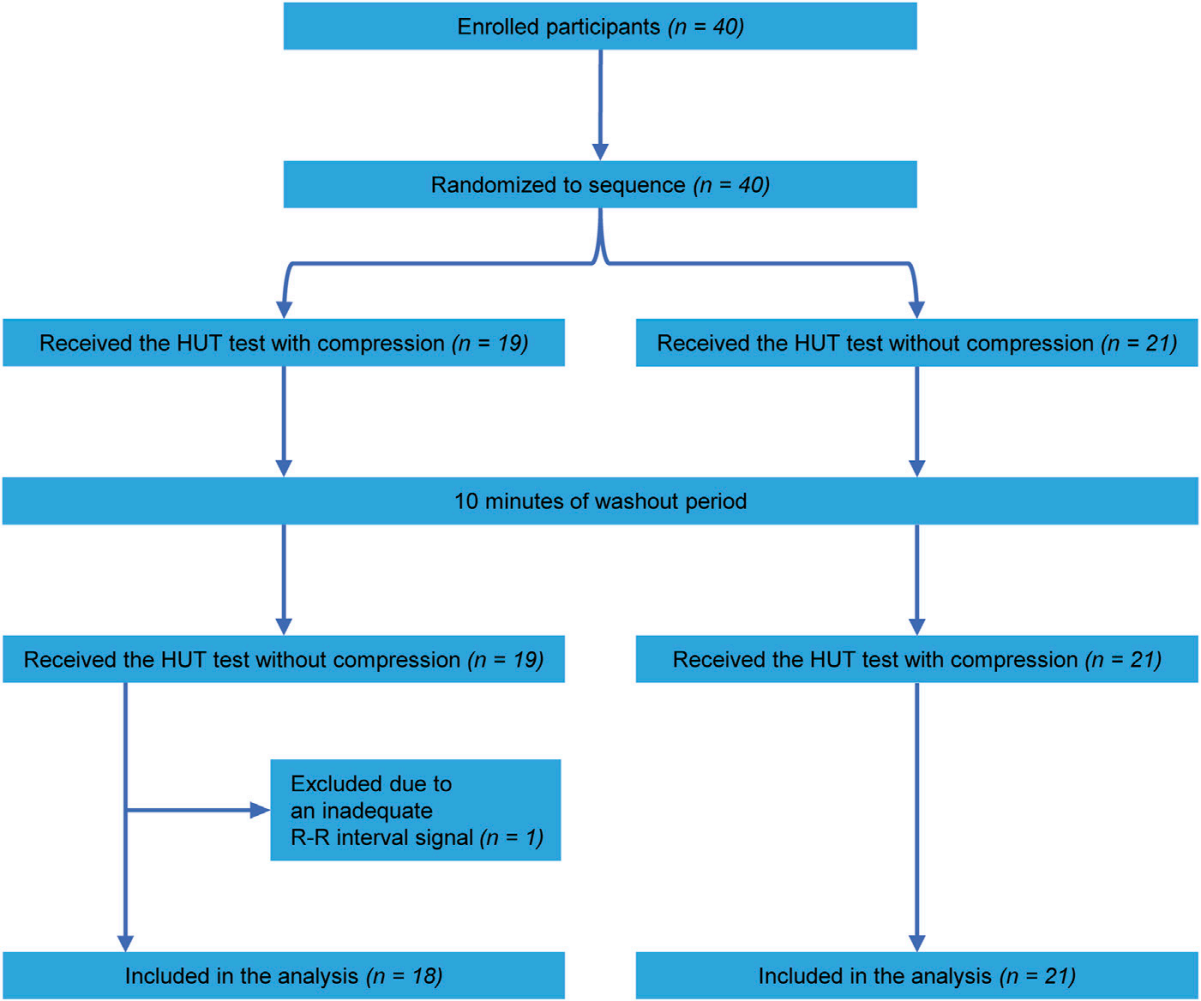
Flow diagram of participant enrollment. HUT, head-up tilt.

A total of 40 healthy volunteers participated in this study. Owing to an inadequate R-R interval signal, one participant was excluded from the analysis. Consequently, data concerning 39 participants were included in the analysis. Table 1 shows the participants’ characteristics. A flow chart of the participants enrolled in this study is shown in Figure 1. According to the randomized sequence, 18 participants underwent the compression condition before the no-compression condition, whereas 21 participants experienced the no-compression condition preceding the compression condition.

**TABLE 1:** Participants’ characteristics.

Variable	Overall (*n* = 39)	Male (*n* = 19)	Female (*n* = 20)
Age (years)	20.9 ± 1.2	21.5 ± 1.4	20.5 ± 0.6
Height (m)	1.65 ± 0.08	1.71 ± 0.05	1.59 ± 0.05
Weight (kg)	55.6 ± 8.5	61.0 ± 6.9	50.5 ± 6.4
Body mass index (kg/m^2^)	20.4 ± 2.1	21.0 ± 2.0	19.9 ± 2.2

Values are presented as the mean ± standard deviation.

### 3.2 Comparisons of hemodynamic and heart rate variability responses to HUT with and without abdominal and lower-extremity compression

**FIGURE 2:**
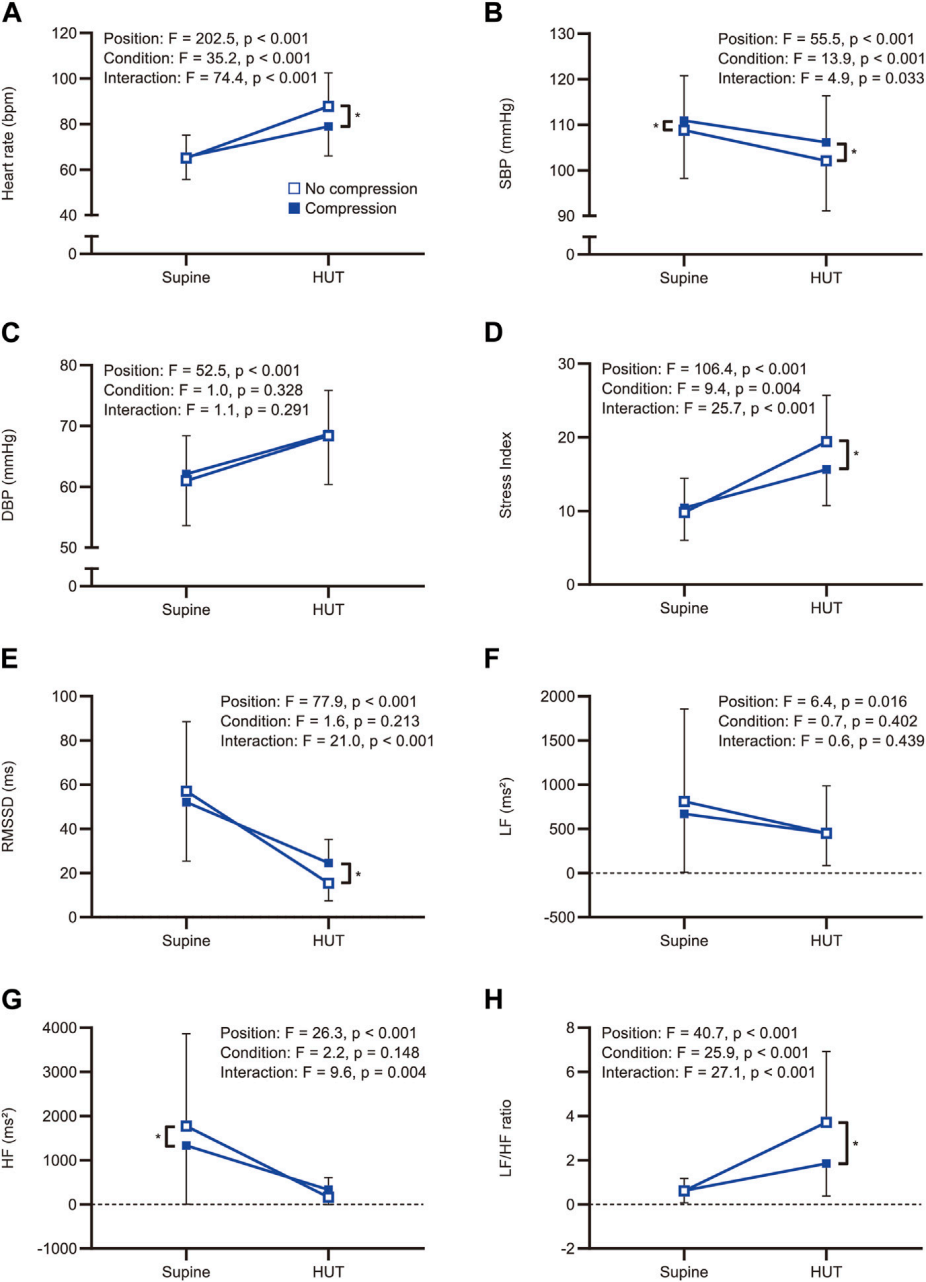
Comparisons of the hemodynamic and heart rate variability responses to head-up tilt with and without compression. Hemodynamic and heart rate variability responses to head-up tilt including **(A)** heart rate, **(B)** systolic blood pressure, **(C)** diastolic blood pressure, **(D)** Stress Index, **(E)** RMSSD, **(F)** LF, **(G)** HF, and **(H)** LF/HF ratio. An asterisk indicates a significant difference between the conditions by the Bonferroni multiple comparison test (*p* < 0.05). HUT, head-up tilt; SBP, systolic blood pressure; DBP, diastolic blood pressure; RMSSD, root mean square of successive differences between adjacent R-R intervals; LF, low-frequency component of heart rate variability; HF, high-frequency component of heart rate variability; LF/HF ratio, low-to-high frequency ratio.

All participants completed a 10-min HUT in both the no-compression and compression conditions without substantial adverse events. The median (interquartile range) VAS scores in the compression condition were significantly lower than those in the no-compression condition [10 (3–23) mm vs. 15 (5–41) mm; W = −228, *p* = 0.032]. Figure 2 illustrates hemodynamic and heart rate variability responses to HUT.

#### 3.2.1 Hemodynamic parameters

**FIGURE 3:**
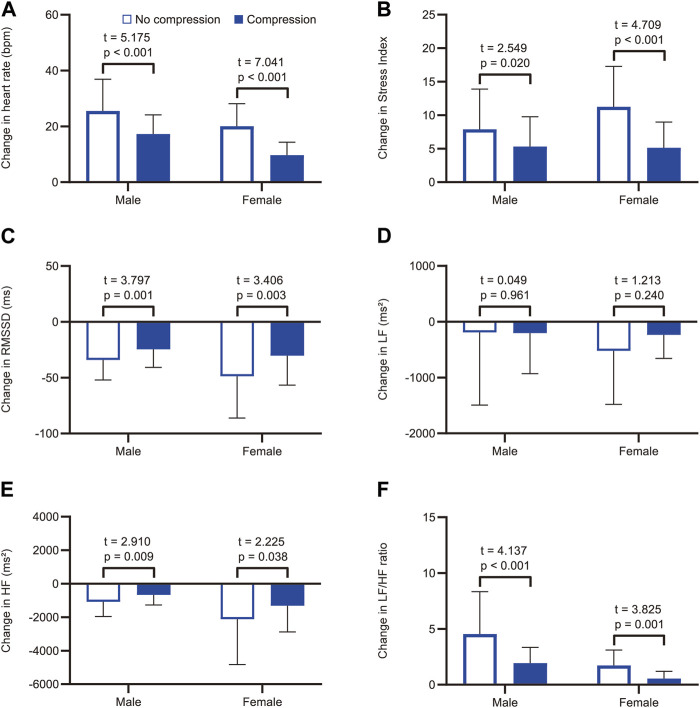
Subgroup analysis by sex for comparisons of the tilt-induced changes in heart rate and heart rate variability parameters with and without compression. Changes in **(A)** heart rate, **(B)** Stress Index, **(C)** RMSSD, **(D)** LF, **(E)** HF, and **(F)** LF/HF ratio from supine to head-up tilt. White and blue bars represent the mean values in the no-compression condition and the compression condition, respectively. RMSSD, root mean square of successive differences between adjacent R-R intervals; LF, low-frequency component of heart rate variability; HF, high-frequency component of heart rate variability.

Representative data of heart rate changes during the HUT test in the no-compression and compression conditions are shown in Supplementary Figure S1. The two-way repeated-measures ANOVA revealed significant main effects for position (F_(1, 38)_ = 202.5, *p* < 0.001) and condition (F_(1, 38)_ = 35.2, *p* < 0.001) on heart rate, as well as a significant interaction between position and condition (F_(1, 38)_ = 74.4, *p* < 0.001; Figure 2A). Post-hoc pairwise comparisons revealed that there was no significant difference in supine heart rates between the conditions (t = 0.687, *p* = 0.993), while the heart rate during the HUT position was significantly lower in the compression condition than in the no-compression condition (t = 11.510, *p* < 0.001). These results indicate that the increase in heart rate during the HUT test was significantly smaller in the compression condition than in the no-compression condition. Six participants (five males and one female) showed a heart rate increase of ≥30 bpm from supine to HUT in the no-compression condition, while no participants exhibited an orthostatic heart rate increase of ≥30 bpm in the compression condition. Subgroup analyses revealed that the increase in heart rate during the test was significantly smaller in the compression condition than in the no-compression condition, regardless of sex (*p* < 0.001; Figure 3A). In addition, the supplemental analysis also indicated a significantly smaller heart rate increase from supine to HUT in the compression condition than in the no-compression condition, regardless of whether the no-compression or compression condition occurred first (*p* < 0.001; Supplementary Figure S2).

Regarding blood pressure variables, there was a statistical main effect of position on systolic (F_(1, 38)_ = 55.5, *p* < 0.001) and diastolic (F_(1, 38)_ = 52.5, *p* < 0.001) blood pressure. For systolic blood pressure, the main effects of the condition (F_(1, 38)_ = 13.9, *p* < 0.001) and the position × condition interaction (F_(1, 38)_ = 4.9, *p* = 0.033) were also significant (Figure 2B), indicating that the reduction in systolic blood pressure was significantly smaller in the compression condition than that in the no-compression condition. In contrast, the main effects of condition (F_(1, 38)_ = 1.0, *p* = 0.328) and the position × condition interaction (F_(1, 38)_ = 1.1, *p* = 0.291) for diastolic blood pressure were not significant (Figure 2C).

#### 3.2.2 Heart rate variability parameters

The two-way repeated-measures ANOVA yielded significant main effects for position (F_(1, 38)_ = 106.4, *p* < 0.001) and condition (F_(1, 38)_ = 9.4, *p* = 0.004) on the Stress Index, as well as a significant interaction between position and condition (F_(1, 38)_ = 25.7, *p* < 0.001; Figure 2D). Regarding RMSSD, there was a significant main effect for position (F_(1, 38)_ = 77.9, *p* < 0.001) and a significant interaction between position and condition (F_(1, 38)_ = 21.0, *p* < 0.001); however, the main effect of condition was not significant (F_(1, 38)_ = 1.6, *p* = 0.213; Figure 2E). In the supine position, there were no significant differences between the conditions for both Stress Index (t = 1.076, *p* = 0.578) and RMSSD (t = 2.300, *p* = 0.054). During the HUT position, a significantly lower Stress Index (t = 6.091, *p* < 0.001) and a significantly higher RMSSD (t = 4.176, *p* < 0.001) were observed in the compression condition than in the no-compression condition. These results indicate that the increase in Stress Index and the decrease in RMSSD during the HUT test were significantly smaller in the compression condition than in the no-compression condition. Subgroup analyses indicated that the increase in Stress Index (Figure 3B) and the decrease in RMSSD (Figure 3C) during the test were significantly smaller in the compression condition than in the no-compression condition, regardless of sex (*p* < 0.05).

Concerning the frequency-domain parameters of heart rate variability, the main effect of position on LF was significant (F_(1, 38)_ = 6.4, *p* = 0.016), whereas the main effects of condition (F_(1, 38)_ = 0.7, *p* = 0.402) and the interaction between position and condition (F_(1, 38)_ = 0.6, *p* = 0.439) were not significant (Figure 2F). In the subgroup analysis, the orthostatic change in LF was not significantly different between the conditions, regardless of sex (*p* > 0.05; Figure 3D). For HF, the main effects of position (F_(1, 38)_ = 26.3, *p* < 0.001) and the position × condition interaction (F_(1, 38)_ = 9.6, *p* = 0.004) were significant, although there was no significant main effect of condition (F_(1, 38)_ = 2.2, *p* = 0.148; Figure 2G). A significantly smaller HF in the compression condition than in the no compression condition was observed during the supine position (t = 3.166, *p* = 0.006), while there was no significant difference in HF between the conditions during the HUT position (t = 1.225, *p* = 0.456). Moreover, the results of two-way repeated-measures ANOVA showed a significant main effect of position (F_(1, 38)_ = 40.7, *p* < 0.001), condition (F_(1, 38)_ = 25.9, *p* < 0.001), and the interaction between position and condition (F_(1, 38)_ = 27.1, *p* < 0.001) for the LF/HF ratio (Figure 2H). The LF/HF ratio in the supine position was not significantly different between the conditions (t = 0.046, *p* = 0.999), while that of the HUT position was significantly lower in the compression condition than in the no-compression condition (t = 7.317, *p* < 0.001). Subgroup analyses revealed that the decrease in HF (Figure 3E) and the increase in LF/HF ratio (Figure 3F) during the HUT test were significantly smaller in the compression condition than in the no-compression condition (*p* < 0.05), regardless of sex.

### 3.3 Heart rate variability parameters associated with the reduction in heart rate during the HUT position with abdominal and lower-extremity compression

**FIGURE 4:**
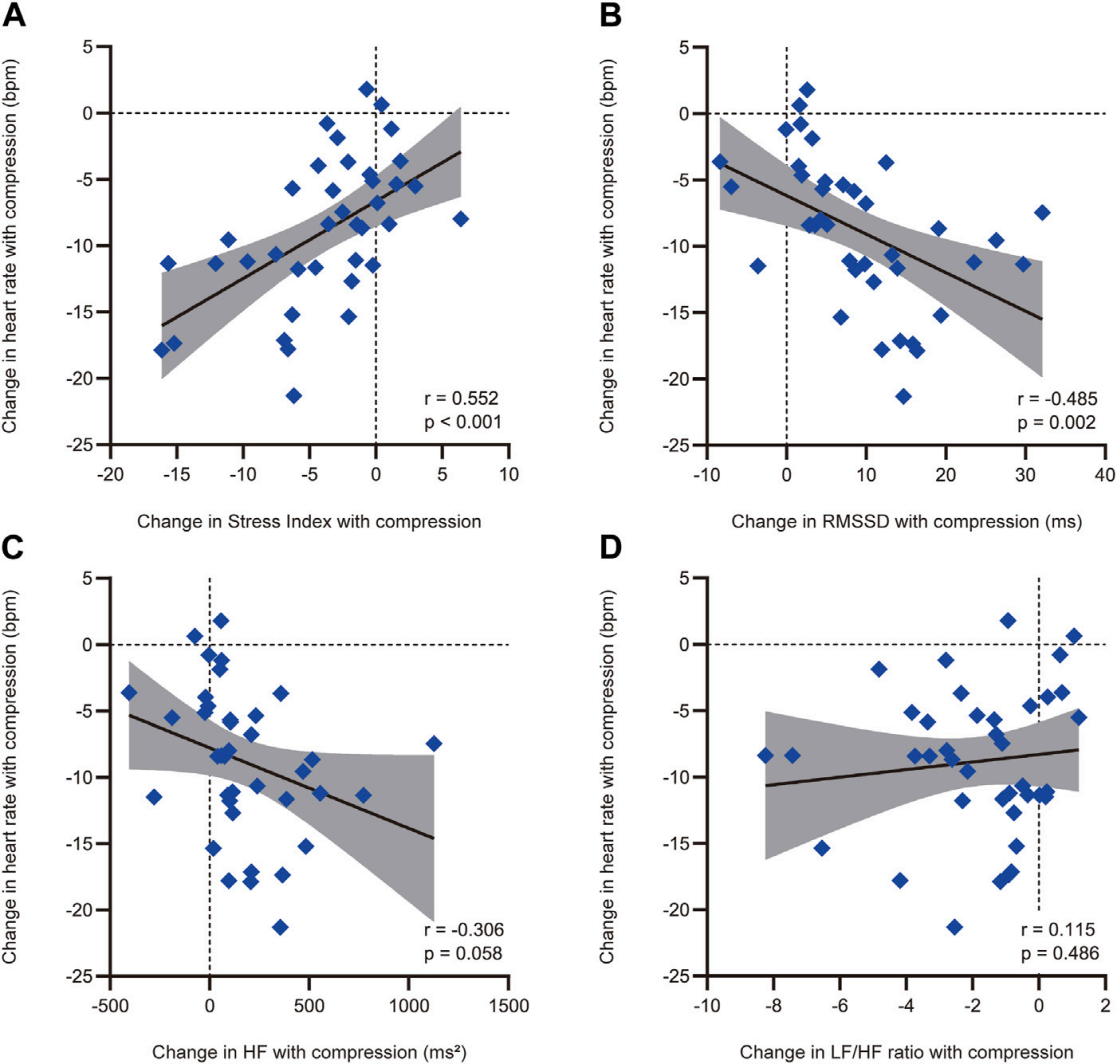
Correlations between heart rate reduction with compression and the compression-induced changes in heart rate variability parameters with a significant difference with and without compression. Correlations between heart rate reduction with compression and compression-induced changes in **(A)** Stress Index, **(B)** RMSSD, **(C)** HF, and **(D)** LF/HF ratio. Heart rate reduction with compression and compression-induced changes in heart rate variability parameters were defined as the differences between the measurements in the head-up tilt position with and without compression. The solid black line represents the regression line, while the shaded area indicates the 95% confidence interval of the regression line. RMSSD, root mean square of successive differences between adjacent R-R intervals; HF, high-frequency component of heart rate variability; LF, low-frequency component of heart rate variability.

The correlations between heart rate reduction with compression and changes in heart rate variability parameters with compression are shown in Figure 4. There was a significant correlation between a greater reduction in heart rate with compression and a larger decrease in Stress Index [r = 0.552, 95% confidence interval (CI) = 0.286 to 0.739, *p* < 0.001; Figure 4A]. Additionally, an increase in RMSSD with compression correlated with a larger heart rate reduction (r = −0.485, 95% CI = −0.695 to −0.200, *p* = 0.002; Figure 4B). However, no significant correlations were found between changes in the HF component (r = −0.306, 95% CI = −0.567 to 0.010, *p* = 0.058; Figure 4C) and the LF/HF ratio (r = 0.115, 95% CI = −0.208 to 0.416, *p* = 0.486; Figure 4D) with compression. The associations between heart rate reduction with compression and potentially confounding variables are presented in Table 2. The greater reduction in heart rate measured during the HUT position with compression was also significantly associated with female sex (mean difference = −4.4, 95% CI = −7.7 to −1.1, *p* = 0.010) but not with other variables (*p* > 0.05). Therefore, the changes in Stress Index and RMSSD with compression and sex were entered in the multiple regression analysis as the independent variables. The results of the multiple regression analysis are shown in Table 3. The analysis (adjusted R^2^ = 0.335, *p* < 0.001) revealed that the decrease in Stress Index with compression was the only significant independent variable for heart rate reduction with compression (coefficient = 0.411, *p* = 0.025).

**TABLE 2:** Associations between heart rate reduction with compression and potential confounding variables.

Variable	Statistics	*p*-value
Age	r = 0.218	0.184
Sex	t = 2.715	0.010
Height	r = 0.181	0.269
Weight	r = 0.199	0.226
Body mass index	r = 0.128	0.439
Change in systolic blood pressure with compression	r = 0.031	0.851
Change in diastolic blood pressure with compression	r = 0.258	0.113

The heart rate reduction and the change in systolic and diastolic blood pressure with compression were defined as the differences between the measurements in the head-up tilt position with and without compression.

**TABLE 3:** Multiple regression analysis to determine heart rate reduction with compression.

Variable	Coefficient	Standard error	t-value	*p*-value
Decrease in Stress Index with compression	0.411	0.175	2.343	0.025
Increase in RMSSD with compression	−0.096	0.103	0.931	0.358
Sex (male = 0, female = 1)	−2.527	1.555	1.625	0.113
Constant	−5.119	1.131	4.526	<0.001
F (3, 35) = 7.4, R^2^ = 0.388, Adjusted R^2^ = 0.335, *p* < 0.001				

Heart rate reduction, decrease in Stress Index, and increase in RMSSD, with compression were defined as the differences between the measurements in the head-up tilt position with and without compression.

RMSSD, root mean square of successive differences between adjacent R-R intervals.

## 4 Discussion

Consistent with previous studies conducted on healthy adults (Watanuki and Murata, 1994; Dorey et al., 2018; Lee et al., 2018; Kelly et al., 2019), our study revealed that abdominal and lower-extremity compression effectively reduced the degree of heart rate elevation during the HUT test. Although several studies have shown the effects of compression on orthostatic changes in hemodynamic variables (Watanuki and Murata, 1994; Denq et al., 1997; Smit et al., 2004; Podoleanu et al., 2006; Platts et al., 2009; Stenger et al., 2010; Stenger et al., 2013; Heyer, 2014; Dorey et al., 2018; Lee et al., 2018; Kelly et al., 2019; Bourne et al., 2021), to the best of our knowledge, this study is the first to compare changes in heart rate variability parameters during the HUT test with and without abdominal and lower-extremity compression in healthy individuals. We found that the orthostatic increases in the Stress Index and LF/HF ratio and the orthostatic decreases in RMSSD and HF were smaller in the compression condition than in the no-compression condition. The reductions in heart rate and alternations in heart rate variability responses to the HUT test with compression were consistent across participants regardless of their sex. In addition, the compression-induced decrease in Stress Index during the HUT position was a significant independent variable for the compression-induced reduction in heart rate in the HUT position. These results may provide insights into the mechanisms underlying the improvements in an excessive orthostatic heart rate increase with compression. The pathophysiological mechanisms underlying POTS are thought to be heterogeneous (Benarroch, 2012; Vernino et al., 2021). Therefore, the findings of this study could contribute to the appropriate application of compression therapy according to the underlying pathophysiology of POTS.

A crossover design allows a participant’s response to one treatment to be contrasted with the same participant’s response to another. Removing participant variation in this way makes crossover trials potentially more efficient than similar-sized, parallel-group trials in which each participant is exposed to only one treatment (Sibbald and Roberts, 1998). In the present study, the HUT tests with and without compression were performed randomly, which may help reduce order effects. We used an inflatable abdominal band with a pressure of 35–45 mmHg and medical compression stockings with a pressure of approximately 35–45 mmHg at the ankles. A study of healthy adults reported that low-pressure sports compression tights with a pressure of 15.2 ± 7.2 mmHg at the calf and 8.5 ± 1.5 mmHg at the thigh attenuated the increase in heart rate during the HUT test (Lee et al., 2018). Studies of individuals with POTS also demonstrated that abdominal and lower-extremity compression with a pressure of 20–40 mmHg reduced orthostatic tachycardia during the HUT test (Heyer, 2014; Bourne et al., 2021). Given the results of these previous studies, the compression pressure used in this study might be sufficient to reduce the orthostatic increase in heart rate.

Heart rate and heart rate variability responses to HUT observed in the no-compression condition support the results of previous studies that performed the HUT test in healthy individuals (Stewart, 2000; Freitas et al., 2015; Orjatsalo et al., 2020; Ali et al., 2021). Similar observations were also reported in studies using a lower body negative pressure (Cooke et al., 2008; Tigges et al., 2019). Lower body negative pressure is a technique that reduces venous return to the heart by causing blood pooling in the lower body, without other effects induced by transitioning from supine to upright, such as altered stimulus patterns in otolithic receptors or full orthostatic weight loading on the lower extremities (Goswami et al., 2019). Therefore, hemodynamic and heart rate variability responses to HUT in the no-compression condition suggest that an orthostatic reduction in venous return to the heart leads to baroreflex-mediated compensatory sympathetic activation, reduced parasympathetic activation, and ultimately increased heart rate. Incidentally, six participants showed an orthostatic heart rate increase of ≥30 bpm in the no-compression condition. Our supplemental analysis indicated that they had significantly more severe orthostatic symptoms [VAS score = 54 (31–83)] than the other 33 participants [VAS score = 10 (4–27)] in the no-compression condition (Mann-Whitney U = 26, *p* = 0.003). Nevertheless, as these six participants had not been diagnosed with POTS, their orthostatic heart rate increase might be an upper limit response in the physiological range of healthy individuals.

The decrease in systolic blood pressure during the HUT test was smaller in the compression condition than in the no-compression condition, which may mean that the venous return to the heart was improved by compression (Watanuki and Murata, 1994; Dorey et al., 2018; Lee et al., 2018). Therefore, the reduction in heart rate and heart rate variability responses to HUT with compression observed in this study suggest that abdominal and lower-extremity compression mechanically displaces blood pooled in the abdomen and lower extremities back to the heart, reducing sympathetic activation and vagal withdrawal. As a result, it attenuates the heart rate increase during the transition from supine to standing positions. These changes in hemodynamic and autonomic responses to HUT might result in the reduction of orthostatic symptoms assessed by VAS (Heyer, 2014; Bourne et al., 2021). In addition, the results of our subgroup analysis indicate that abdominal and lower-extremity compression is likely to reduce orthostatic responses of heart rate and heart rate variability parameters, irrespective of sex. As no participants had been diagnosed with POTS, future studies are warranted to evaluate the effectiveness of compression garments in males with POTS.

The results of the correlation analysis supported that the compression-induced decrease in cardiac sympathetic activity and increase in vagal activity during the HUT position were related to the compression-induced heart rate reduction in the HUT position. In addition, the results of multiple regression analysis suggest that a decrease in cardiac sympathetic activity, rather than an increase in vagal activity, is associated with the heart rate reduction in the HUT position with compression. Excessive central sympathetic activation is one of the possible mechanisms leading to POTS, which is called hyperadrenergic POTS (Lambert and Lambert, 2014; Sheldon et al., 2015; Arnold et al., 2018; Bryarly et al., 2019; Vernino et al., 2021). Central sympatholytic agents, such as clonidine and methyldopa, improve orthostatic tachycardia in individuals with hyperadrenergic POTS (Sheldon et al., 2015; Vernino et al., 2021). However, non-pharmacological therapies are often recommended as a first-line treatment for POTS (Sheldon et al., 2015; Fu and Levine, 2018; Bryarly et al., 2019; Vernino et al., 2021). Considering our results of multiple regression analysis, abdominal and lower-extremity compression may be a beneficial treatment for individuals with hyperadrenergic POTS. Further studies are needed to examine whether abdominal and lower-extremity compression can reduce these individuals’ excessive central sympathetic activity and orthostatic tachycardia.

This study had some limitations. First, our supplemental analysis showed a statistically significant but slight decrease in supine heart rate during the second HUT test than observed in the first test (mean difference = −2.5, 95% CI = −4.1 to −0.9, *p* = 0.003), although there were no significant differences in heart rate variability parameters during the supine period between the two HUT tests (Supplementary Table S1). These results suggest that an effect of vagal rebound at the end of the first HUT test was still present during the supine period of the second HUT test. As at least 10 min of rest in the supine position is considered necessary to establish stable baseline hemodynamics (Cheshire and Goldstein, 2019; Finucane et al., 2019), we set a 10-min supine rest period as a washout period. However, the fluid redistribution from standing to supine is slow (Scharfetter et al., 1997). Additionally, an approximately 30-min supine rest period is recommended before the start of autonomic nervous system function testing (Zygmunt and Stanczyk, 2010). As a short washout period may introduce some bias, our results should be interpreted with caution. Second, we evaluated only healthy adults to demonstrate normal compression-induced changes in heart rate variability responses to HUT. Further studies of individuals with POTS are warranted to confirm the robustness of our findings. Third, blinding participants to the compression condition was not feasible in our study because of the visible and tangible nature of the inflatable abdominal band and medical compression stockings. Even sham garments without compression would have felt distinct and not allowed for effective blinding. Thus, any placebo effects might have contributed to the results. However, this appears to be consistent with the actual situation of the use of abdominal and lower-extremity compression. Finally, the tests were performed in the evening. Previous research has demonstrated that the orthostatic increase in heart rate tends to be greater in the morning than in the afternoon (Brewster et al., 2012). Given that a higher heart rate increase from supine to HUT in the no-compression condition is associated with a greater heart rate reduction in the HUT position with compression (Bourne et al., 2021), this study may underestimate the effects of compression on heart rate and heart rate variability responses to HUT.

In conclusion, this study demonstrated that the heart rate and heart rate variability responses to HUT were smaller in the compression condition than in the no-compression condition. These results suggest that abdominal and lower-extremity compression attenuates orthostatic cardiac sympathetic activation and orthostatic decrease in vagal activity in healthy adults. In addition, our results also indicate that the decreased cardiac sympathetic activity may be associated with the attenuation of the orthostatic heart rate increase with compression, which could contribute to understanding the mechanisms underlying the improvements of an excessive orthostatic heart rate increase with compression. The findings of this study may have important implications for the application of abdominal and lower-extremity compression to prevent the development of orthostatic tachycardia.

## Data Availability

The raw data supporting the conclusion of this article will be made available by the authors, without undue reservation.
