# Supplementary material for: Comparisons of heart rate variability responses to head-up tilt with and without abdominal and lower-extremity compression in healthy young individuals: a randomized crossover study
Source: Front Physiol. 2024 Jan 8;14:1269079. doi: 10.3389/fphys.2023.1269079 (PMC10800437; doi:10.3389/fphys.2023.1269079)
Supplement: Supplementary file 1 [file DataSheet1.pdf]

Supplementary Material

Supplementary Figures

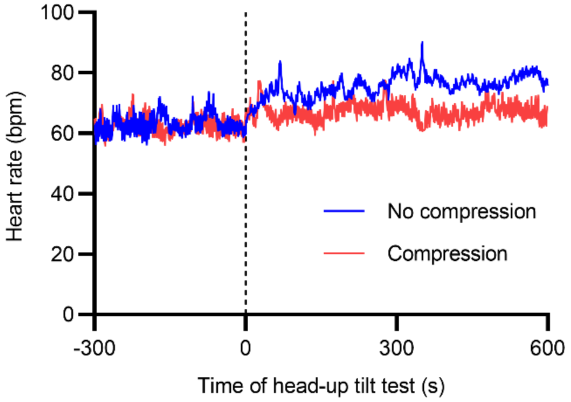

Variable	Position	No compression	Compression
Heart rate (bpm)	Supine	63.5	62.4
	HUT	80.3	68.6
Stress Index	Supine	9.4	11.1
	HUT	22.6	12.9
RMSSD (ms)	Supine	45.9	43.6
	HUT	11.8	35.4
LF (ms^2)	Supine	566	229
	HUT	62	148
HF (ms^2)	Supine	913	790
	HUT	55	609
LF/HF ratio	Supine	0.62	0.29
	HUT	1.13	0.24

Supplementary Figure S1. Spectrum of heart rate responses to head-up tilt with and without compression.

Representative heart rate changes during the head-up tilt test with (red line) and without (blue line) compression in a female participant. The vertical dashed line indicates the start of the postural change. The table below the figure represents the measurement values of heart rate and heart rate variability parameters.
RMSSD, root mean square of successive differences between adjacent R-R intervals; LF, low-frequency component of heart rate variability; HF, high-frequency component of heart rate variability

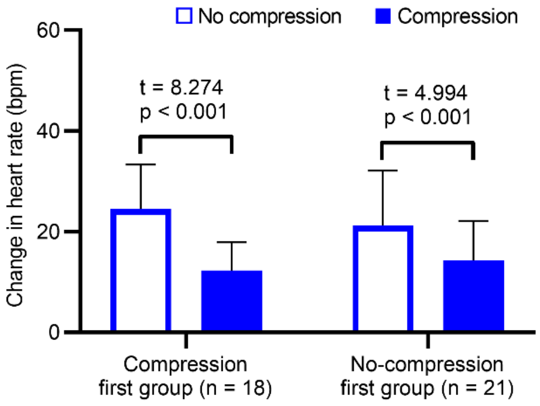

Supplementary Figure S2. Comparison of heart rate changes during the head-up tilt test between the conditions in the compression-first and no-compression-first groups

White and blue bars indicate the mean values of heart rate changes during the head-up tilt test in the no-compression and compression conditions, respectively. Error bars indicate the standard deviation.

Supplementary Table S1. Comparisons of heart rate and heart rate variability parameters during the supine position between the first and second head-up tilt tests

Variable	First HUT test	Second HUT test	t-value	p-value
Heart rate (bpm)	66.6 ± 10.3	64.0 ± 9.6	3.145	0.003
Stress Index	10.4 ± 3.7	9.9 ± 4.1	1.217	0.231
RMSSD (ms)	51.9 ± 26.3	57.3 ± 31.7	1.953	0.058
LF (ms^2)	691 ± 915	785 ± 842	0.582	0.564
HF (ms^2)	1445 ± 1434	1661 ± 2037	1.102	0.278
LF/HF ratio	0.57 ± 0.48	0.67 ± 0.63	1.429	0.161

Values are presented as mean ± standard deviation.
HUT, head-up tilt; RMSSD, root mean square of successive differences between adjacent R-R intervals; LF, low-frequency component of heart rate variability; HF, high-frequency component of heart rate variability
